# Supplementary material for: Venomix: a simple bioinformatic pipeline for identifying and characterizing toxin gene candidates from transcriptomic data
Source: PeerJ. 2018 Jul 31;6:e5361. doi: 10.7717/peerj.5361 (PMC6074769; doi:10.7717/peerj.5361)
Supplement: Supplemental Information 3 [file peerj-06-5361-s003.gz › FinalOutput_E-20/Putative_protein-glutamate_O-methyltransferase_1/finaltree.pdf]

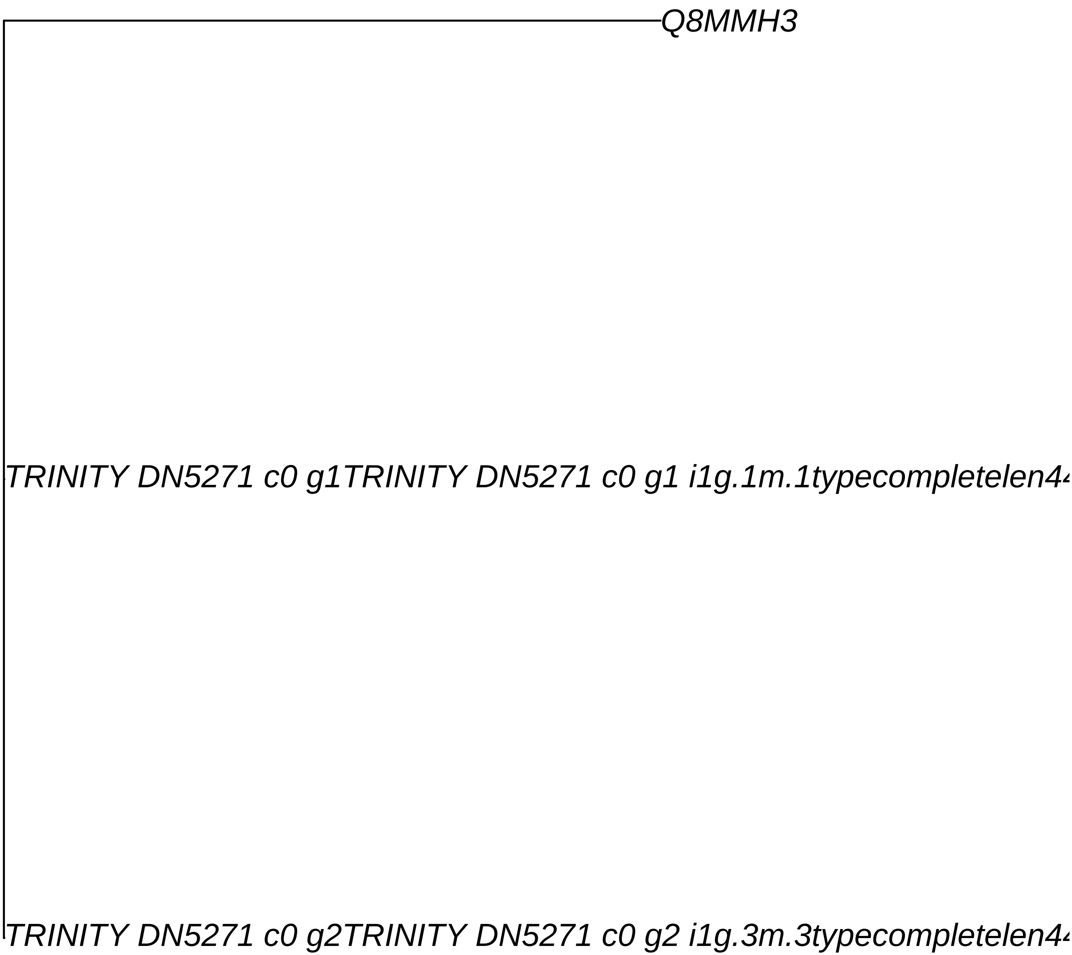
Q8MMH3
TRINITY DN5271 c0 g1TRINITY DN5271 c0 g1 i1g.1m.1typecompletelen44
TRINITY DN5271 c0 g2TRINITY DN5271 c0 g2 i1g.3m.3typecompletelen44
